# Composition and driving factors of arbuscular mycorrhizal fungal communities in the roots and rhizosphere soil of naturally regenerated *Phoebe bournei* seedlings in Guizhou Province, China

**DOI:** 10.1128/spectrum.00210-25

**Published:** 2025-07-07

**Authors:** Xian Liang, Xinyuan Lu, Yi Wei, Fuyin Jiang, Mingbin Wang, Xiaoli Wei

**Affiliations:** 1 College of Forestry, Guizhou University 662625, Guiyang, China; 2 Institute for Forest Resources and Environment of Guizhou, Guizhou University 71206, Guiyang, China; Instituto de Ecología, A.C. (INECOL), Michoacán, Mexico

**Keywords:** *Phoebe bournei*, arbuscular mycorrhizal fungi, soil property, community composition, driving factor

## Abstract

Arbuscular mycorrhizal (AM) fungi play vital roles in promoting tree growth and maintaining biodiversity and ecosystem stability in subtropical forests. *Phoebe bournei*, a key species endemic to the subtropical evergreen broad-leaved forests of China, forms symbiosis associations with AM fungi. However, the composition and structure of AM fungal communities associated with naturally regenerated *P. bournei* remain insufficiently characterized. This study used Illumina MiSeq sequencing to investigate the AM fungal communities in the root and rhizosphere soil samples. In total, 305 operational taxonomic units (OTUs) belonging to four orders and seven families were uncovered within Glomeromycota. Seven and nine AM fungal genera were detected in root and rhizosphere soil samples, respectively, with *Glomus* being the most dominant genus in both root and rhizosphere soil samples. Moreover, the diversity of AM fungal communities varied across sampling locations in the rhizosphere soil and roots. The co-occurrence network structure of the AM fungal community in the rhizosphere soil was more complex and robust than that of the roots. Furthermore, soil properties, latitude, and altitude influenced the changes in AM fungal α-diversity and the relative abundance of genera in roots and rhizosphere soil to varying degrees. Overall, our findings highlight the pivotal role of soil properties over geographical variables in explaining variations in the AM fungal community structure, with soil properties—particularly total phosphorus and total nitrogen—markedly driving the AM fungal community structure in the rhizosphere soil and roots of naturally regenerated *P. bournei* seedlings.

**IMPORTANCE:**

Although subtropical forest ecosystems harbor rich arbuscular mycorrhizal (AM) fungal resources, insights into their communities in the rhizosphere of *Phoebe bournei* remain limited. This study investigates the composition and key drivers of AM fungi communities in the rhizosphere soil and roots of naturally regenerated *P. bournei* seedlings in Guizhou, subtropical China. The findings deepen the understanding of the potential of AM fungi in supporting the establishment and growth of mycorrhizal plants, as well as maintaining the diversity, productivity, and stability of subtropical forest ecosystems. Moreover, this study provides valuable insights into the selection and application of AM fungi resources in mycorrhizal seedling cultivation and afforestation of *P. bournei*.

## INTRODUCTION

Arbuscular mycorrhizal (AM) fungi (Glomeromycota) are widespread in terrestrial ecosystems, forming mutualistic associations with many terrestrial plants (1). These fungi facilitate nutrient uptake through hyphal networks (2), while the host plants provide carbohydrates and lipids necessary for fungal survival (3, 4). AM fungi also improve host plant resistance to abiotic stresses and pathogens (5), and influence plant productivity, competition, and community structure (6). The functioning of AM fungi in plant performance and ecosystem processes is closely linked to the community composition and diversity (7). Assessing AM fungal diversity and community composition provides additional insights into their varying ecological roles.

Consequently, a growing number of studies aim to elucidate the mechanisms of AM fungal community assembly and identify the factors driving their variability. Abiotic environmental factors (e.g., soil properties, latitude, altitude, and climate) and biotic factors (e.g., host plant identity, plant community composition, and species richness) represent the two primary categories influencing AM fungal distribution (8). Low host plant–AM fungal specificity has been reported (9, 10). Environmental factors are dominant drivers structuring the AM fungal community, ranging from regional to global scales (11–13). Among these, geographical variables and soil properties play critical roles in driving AM fungal distribution and community composition (14–16). Altitudinal and latitudinal gradients can influence the distribution and diversity of AM fungal taxa. For instance, on Mount Mila in the Tibetan Plateau, AM fungal communities exhibited potential niche differentiation along altitudinal gradients, with the taxon diversity decreasing with increasing altitude on the eastern slope (17). Furthermore, AM fungal diversity peaked at mid-altitude and then decreased with increasing altitude (18). Ma et al. found that AM fungal richness and Shannon index declined with increasing latitude at a global scale (19). Additionally, soil pH and nutrients were demonstrated to be the key determinants in shaping AM fungal taxon distribution and community structure (11, 19, 20).

Subtropical forests in China have a high diversity of tree species (21) and AM fungi (22), both of which contribute markedly to ecosystem functioning and stability (5, 23). *Phoebe bournei*, a species endemic to China, is one of the key tree species in subtropical evergreen broad-leaved forests (24, 25). Seedling regeneration is crucial for the self-recovery of *P. bournei* in subtropical forest ecosystems. Tree seedlings play an important role in determining community composition and stand structure (26) and in maintaining forest ecosystem stability (27). Beyond its ecological role, *P. bournei* is a precious timber species, known as “wood with golden wire,” for its fine structure, beautiful wood texture, aromatic durability, and high resistance to decay (25). Owing to its significant ecological and economic value, *P. bournei* is widely used in afforestation efforts across southern China (28). Thus, abundant, high-quality *P. bournei* seedlings are critical for afforestation success.

AM fungi can facilitate the establishment and growth of tree seedlings and are closely associated with the performance of seedlings (29–31). *P. bournei* is a tree species capable of forming symbiosis with AM fungi (32). To date, studies on AM fungal community composition associated with *P. bournei* have been limited to the rhizosphere soil of young plantations in Jiangxi Province (33). AM fungal community composition and structure in the roots and rhizosphere soil of natural *P. bournei* communities—as well as the environmental factors shaping those communities—remain largely unexplored. Furthermore, whether the AM fungi present in the roots and rhizosphere soil of *P. bournei* contribute to seedlings' growth and development remains unclear.

To address these gaps, we employed Illumina MiSeq sequencing to characterize the AM fungal communities in the roots and rhizosphere soil of *P. bournei* seedlings across seven natural communities in the Guizhou Province of subtropical China. Moreover, we investigated the environmental factors influencing AM fungal community structure by analyzing soil properties and geographical variables. The study objectives are as follows: (i) revealing the diversity, community composition, and co-occurrence network of AM fungi in natural *P. bournei* communities, (ii) assessing how AM fungal diversity and community composition respond to environmental factors, and (iii) identifying the key factors driving variations in AM fungal community structure in the roots and rhizosphere soil of *P. bournei*.

## RESULTS

### Rhizosphere soil properties

**Fig 1:**
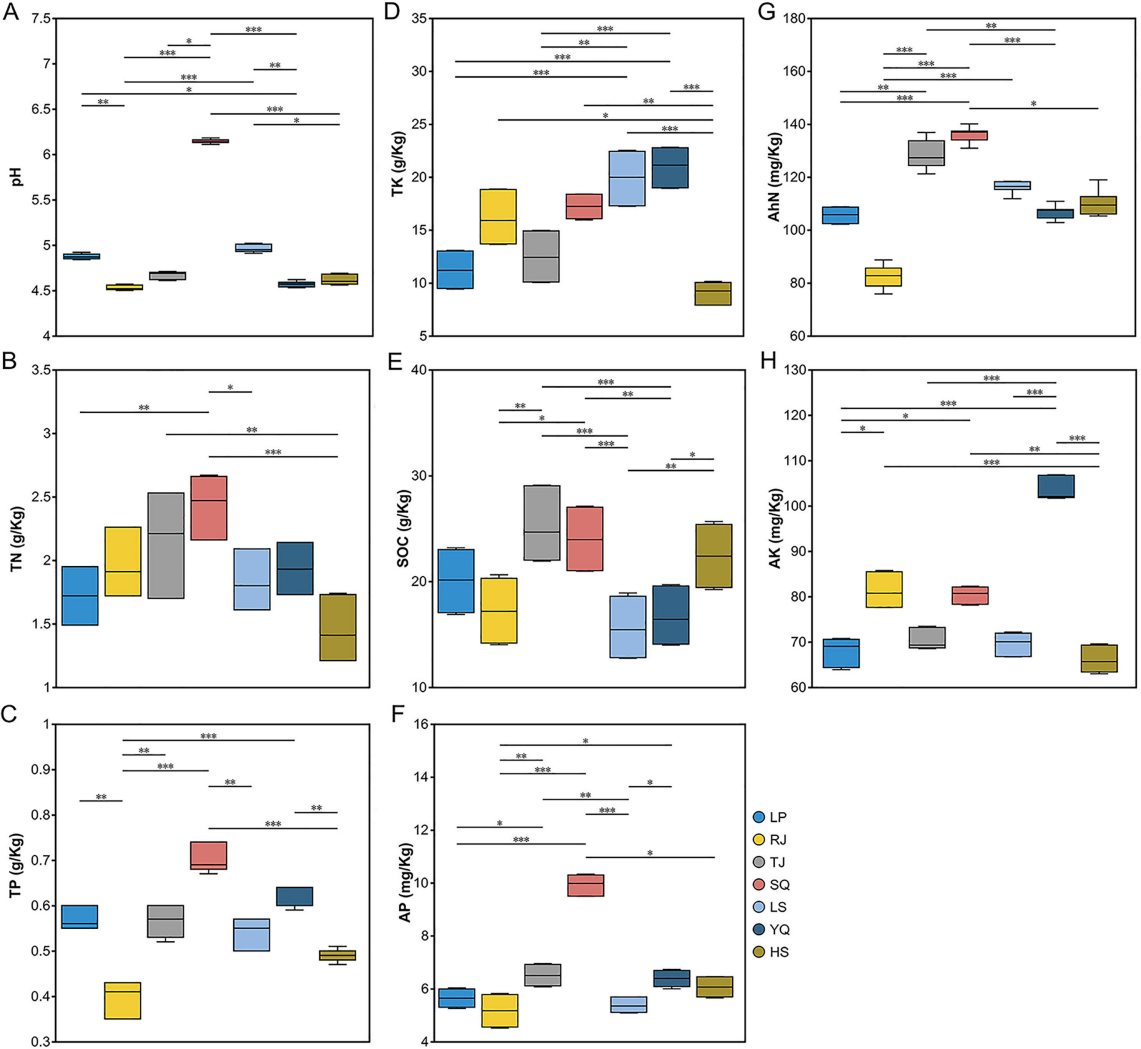
Rhizosphere soil properties of *Phoebe bournei* seedlings from seven sampling locations. Significant differences between sampling locations were determined by the nonparametric Kruskal–Wallis rank sum test. Median (central black line), quartile (box), and minimum and maximum (whiskers) were shown (*n* = 9). * Indicates significant difference, with significance levels of *P < 0.05, **P < 0.01, and ***P < 0.001. LP, RJ, TJ, SQ, LS, YQ, and HS represent Liping, Rongjiang, Taijiang, Shiqian, Leishan, Yuqing, and Huishui, respectively. A: pH; B: TN, total nitrogen; C: TP, total phosphorus; D: TK, total potassium; E: SOC, soil organic carbon; F: AP, available phosphorus; G: AhN, alkaline-hydrolyzable nitrogen; H: AK, available potassium.

We first estimated rhizosphere soil properties from all seven locations. The pH values across all study locations showed acidic soils (4.53–6.14) (Fig. 1A). Among the locations, Shiqian exhibited the highest levels of pH, total nitrogen (TN), total phosphorus (TP), available phosphorus (AP), and alkaline hydrolyzable nitrogen (AhN) in the rhizosphere soil (Fig. 1A, B, C, F, and G). However, the TP, AP, and AhN levels were lowest in Rongjiang (Fig. 1C, F, and G). Soil organic carbon (SOC) content was significantly lower in Leishan and Yuqing than in Huishui, Shiqian, and Taijiang (Fig. 1E). Total potassium (TK) and available potassium (AK) were highest in Yuqing (Fig. 1D and H), whereas Huishui exhibited the lowest TN, TK, and AK values (Fig. 1B, D, and H). These data suggest that the rhizosphere soil properties of the seven study locations were highly heterogeneous.

### AM fungal OTU sequences in roots and rhizosphere soil of *P. bournei* seedlings

Across all analyzed samples, 453,832 and 464,560 high-quality AM fungal sequences were obtained from rhizosphere soil and root samples, respectively, using Illumina MiSeq sequencing. These samples yielded a 216 bp average read length, including 17,793–23,884 AM fungal sequences per sample (mean = 21,866; Table S1). The operational taxonomic unit (OTU) rarefaction curves were saturated for all rhizosphere soil and root samples (Fig. S1), indicating that sampling and sequencing captured most AM fungi taxa in the present study. In total, 305 AM fungal OTUs were detected based on 97% sequence similarity. Among them, 45 and 50 OTUs were unique to roots and the rhizosphere soil, respectively, while 210 OTUs were shared between both compartments of rhizosphere soil and roots (Fig. S2).

### AM fungal community composition

**Fig 2:**
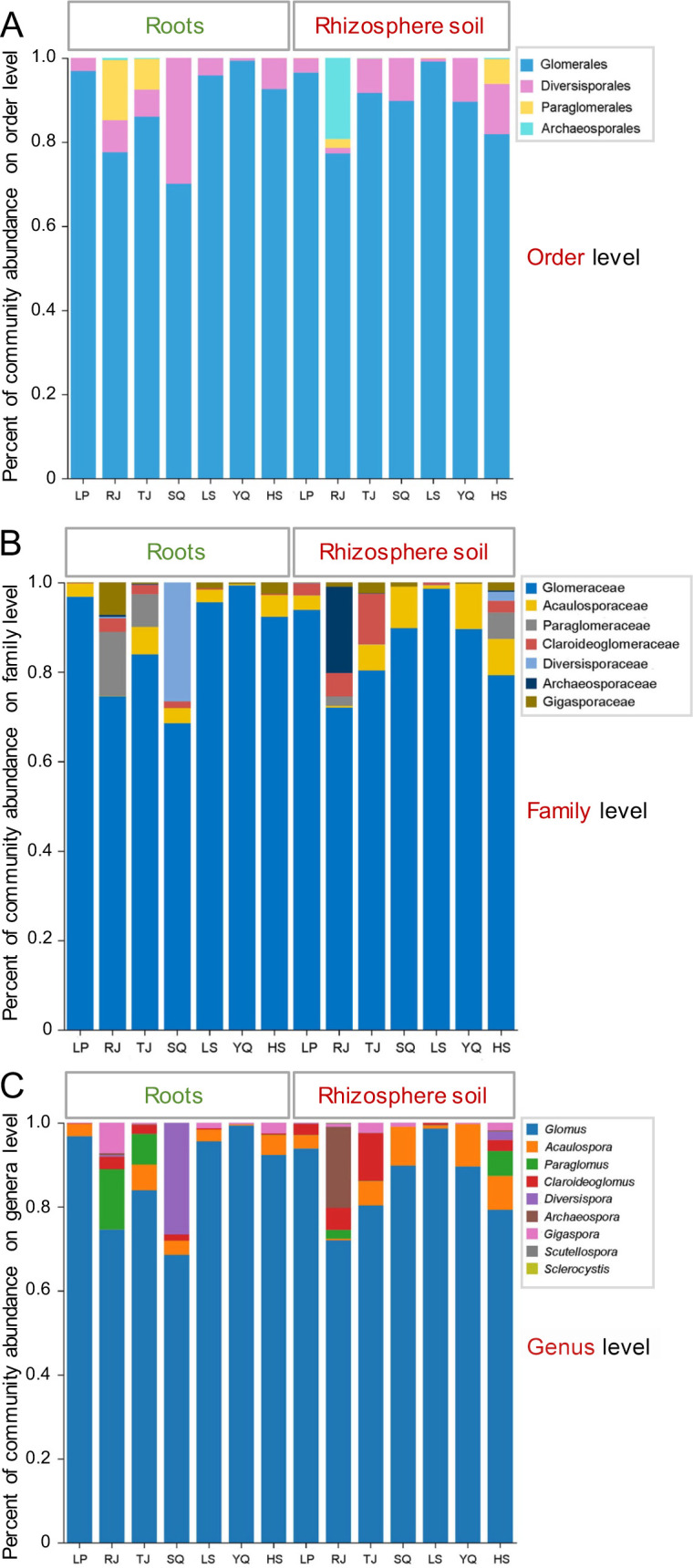
Relative abundance of AM fungal community composition. (**A**) Order, (**B**) family, and (**C**) genus levels in roots (left) and rhizosphere soil (right) of *Phoebe bournei* seedlings, respectively.

The 305 OTUs obtained in the present study were assigned to four orders, seven families, and nine genera of Glomeromycota. Order Glomerales (226 OTUs; 88.88% of sequences) displayed the highest abundance, followed by Diversisporales (33 OTUs; 7.54%), Paraglomerales (15 OTUs; 2.13%), and Archaeosporales (31 OTUs; 1.45%) (Fig. 2A). Of these seven detected families—Glomeraceae, Acaulosporaceae, Paraglomeraceae, Claroideoglomeraceae, Diversisporaceae, Archaeosporaceae, and Gigasporaceae—Glomeraceae, Acaulosporaceae, and Paraglomeraceae displayed the highest relative abundances (86.74%, 4.14%, and 2.13%, respectively) (Fig. 2B). Nine AM fungal genera were identified: *Glomus*, *Acaulospora*, *Paraglomus*, *Claroideoglomus*, *Diversispora, Archaeospora*, *Gigaspora*, *Scutellospora,* and *Sclerocystis* (Fig. 2C). Of these, *Glomus*, *Acaulospora*, *Paraglomus*, *Claroideoglomus*, and *Diversispora* had relative abundance of >1%, jointly accounting for >97% of all sequence reads recovered.

*Glomus* showed the highest abundance in both root and rhizosphere soil samples (mean relative abundances: 86.74%) (Fig. 2C). While most genera were found in all samples from roots and rhizosphere soil, *Scutellospora* and *Sclerocystis* remained undetected in the roots. For the collected root samples, *Glomus* was notably the most abundant genus, representing 87.29% of the total reads, followed by *Diversispora* (3.85%) and *Paraglomus* (3.10%). Moreover, the AM fungal communities were dominated by *Glomus* (86.20%), *Acaulospora* (5.37%), and *Claroideoglomus* (3.22%) from the collected rhizosphere soil samples. *Scutellospora* and *Sclerocystis* appeared only in the rhizosphere soil of Rongjiang and corresponded to the least abundant genus in soil samples (<0.05%).

### AM fungal community diversity

**Fig 3:**
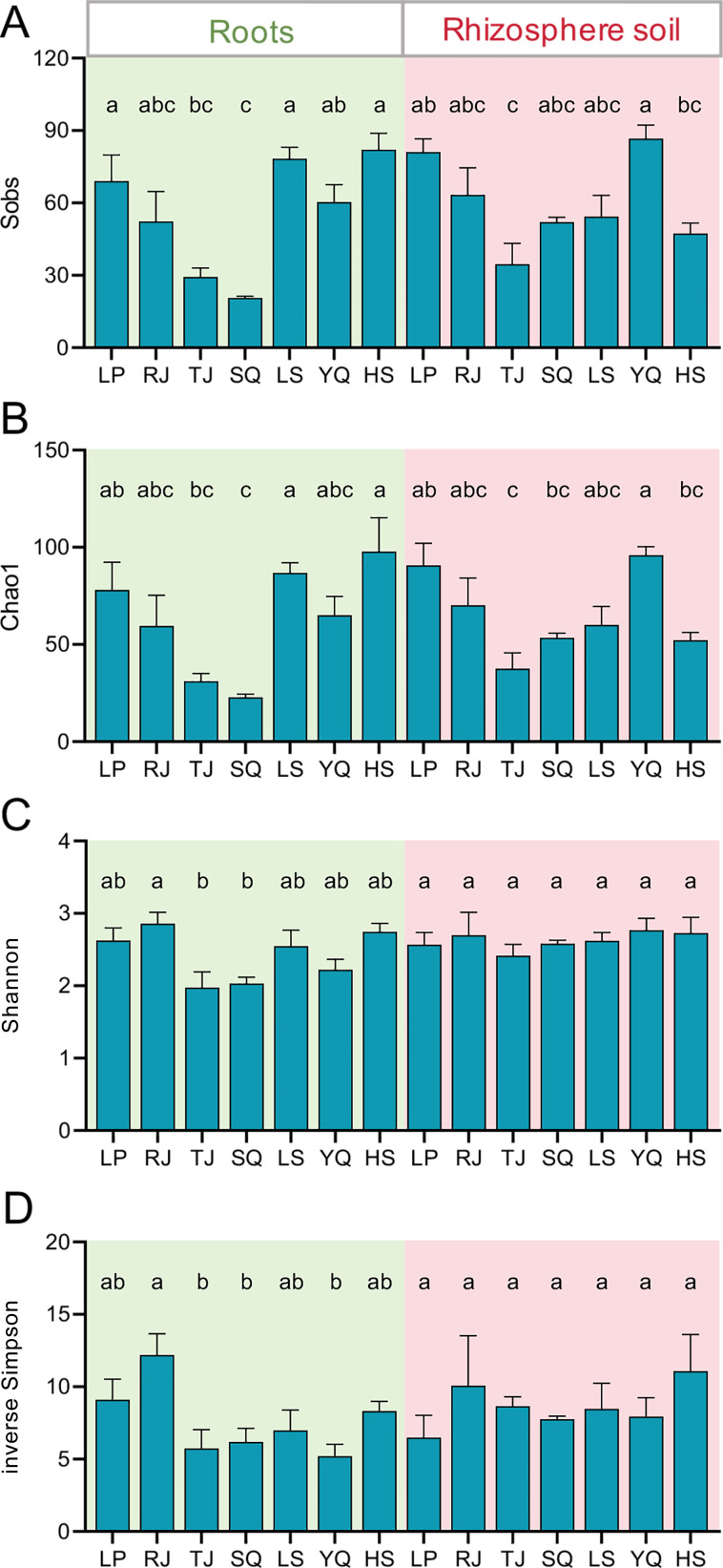
AM fungal α-diversity in roots (left) and rhizosphere soil (right) of *Phoebe bournei* seedlings from different sampling locations. Vertical bars indicate standard error (*n* = 3). Different lowercase letters on each column indicate significant difference at *P* < 0.05 among sampling locations as determined by Tukey’s test. A: Sobs; B: Chao1; C: Shannon; D: inverse Simpson.

The Sobs, Chao1, Shannon, and inverse Simpson indices of AM fungi in the roots showed significant difference among sampling locations (*P* < 0.05; Fig. 3A through D). In rhizosphere soil, Sobs and Chao1 indices showed significant differences among sampling locations (*P* < 0.05) (Fig. 3A and B), unlike Shannon and inverse Simpson indices (*P* > 0.05; Fig. 3C and D). Notably, the Good’s coverage indices for all samples exceeded 0.999, indicating that the AM fungal community composition was well reflected by Illumina sequencing (Table S1).

**Fig 4:**
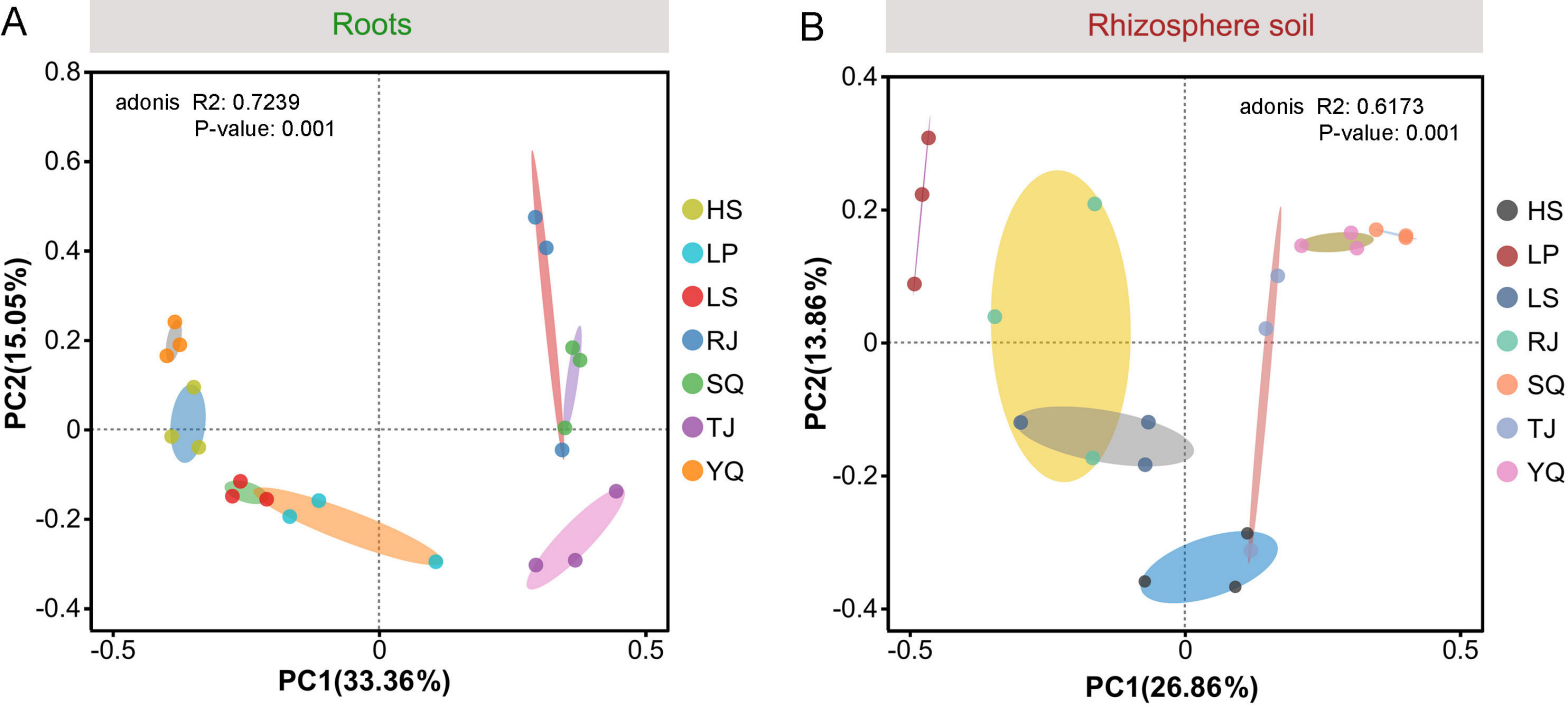
PCoA analysis of AM fungal communities at the OTU level based on Bray–Curtis distances in the roots (**A**) and rhizosphere soil (**B**) of *Phoebe bournei* seedlings from different sampling locations. PERMANOVA demonstrated the significance and the variance(R^2^) of differentiation among AM fungal communities from the seven sampling locations. LP, RJ, TJ, SQ, LS, YQ, and HS represent Liping, Rongjiang, Taijiang, Shiqian, Leishan, Yuqing, and Huishui, respectively.

As shown in the principal coordinate analysis (PCoA) plots, the AM fungal community composition in the collected root and rhizosphere soil samples exhibited variations among the sampling locations (Fig. 4A and B). Furthermore, permutational multivariate analysis of variance (PERMANOVA) demonstrated significant dissimilarity in AM fungal community structures in both samples across different sampling locations (roots: R^2^ = 0.7239, *P* = 0.001; rhizosphere soil: R^2^ = 0.6173, *P* = 0.001) (Fig. 4A and B).

### Co-occurrence patterns associated with AM fungal communities in roots and rhizosphere soil

**Fig 5:**
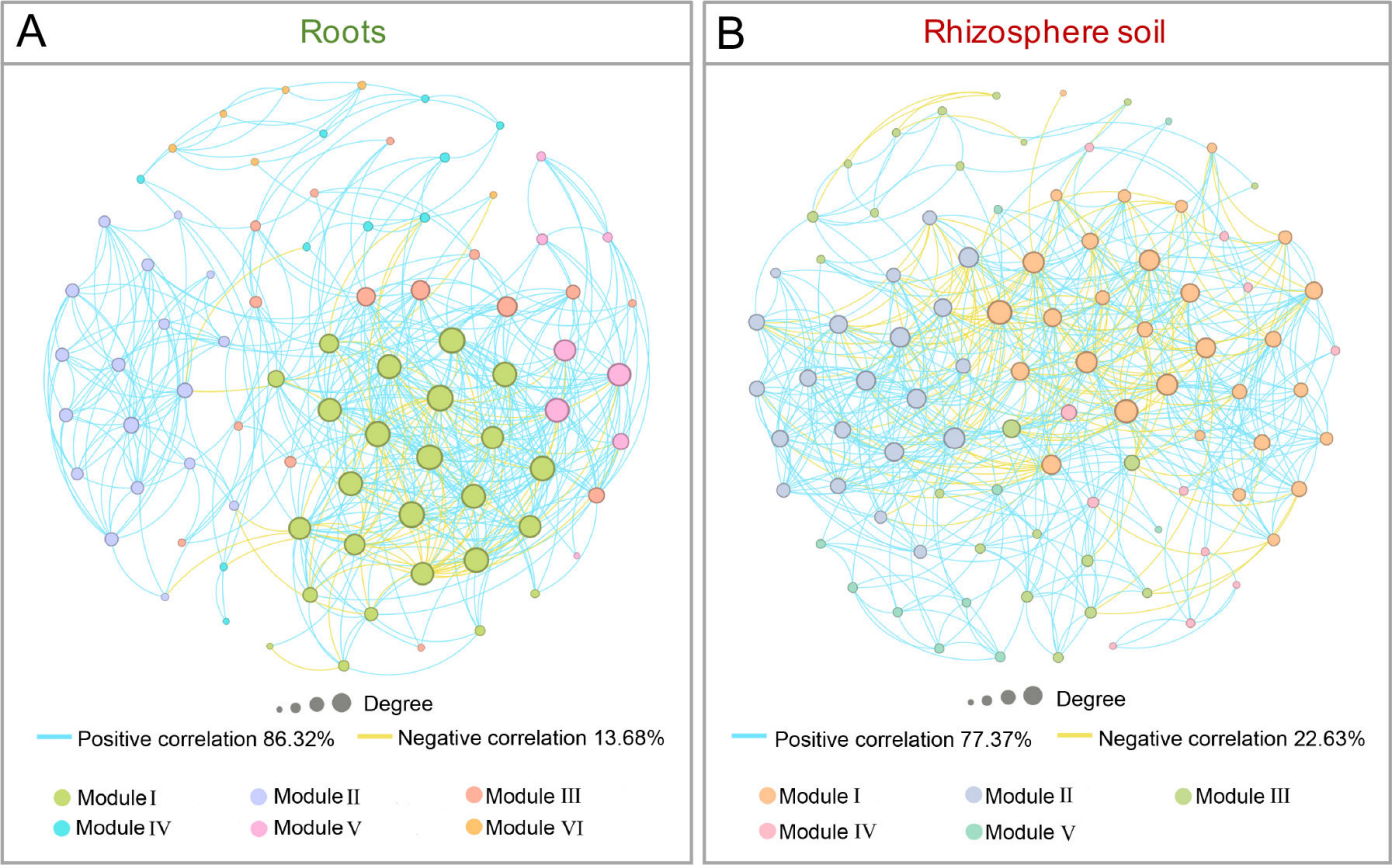
Co-occurrence networks of AM fungi in the roots (**A**) and rhizosphere soil (**B**) of *Phoebe bournei* seedlings. Significant associations (|r| > 0.50, *P* < 0.05) between OTUs were observed. Each node represents an AM fungi OTU. The node size represents the degree of the node, and nodes with the same color display the same module. Edges represent correlations between nodes: positive correlations are shown in blue and negative correlations are shown in yellow.

The root network had fewer nodes and edges (82 nodes and 475 edges) (Fig. 5A) than in rhizosphere soil network (93 nodes and 570 edges) (Fig. 5B). The average degrees of root and rhizosphere soil were 11.59 and 12.26, respectively. The average clustering coefficient was 0.59 in roots and 0.56 in rhizosphere soil. The modularity index of the rhizosphere soil AM fungi (0.44) exceeded that of the root AM fungal communities (0.40). The average path length between nodes of AM fungal communities in roots (2.88) surpassed that in rhizosphere soil (2.60). In addition, the proportion of negative correlations existing in the AM fungal co-occurrence network of the rhizosphere soil exceeded those of the roots (22.63% vs 13.68%). These findings suggest that AM fungal co-occurrence networks in the roots and rhizosphere soil display distinct co-occurrence patterns.

### Influence of soil properties and geographical variables on AM fungal diversity and community composition

Correlations of soil properties and geographical variables with AM fungal richness and diversity differed between the roots and rhizosphere soil (Table 1). In roots, SOC and soil TN were significantly negatively correlated with the Sobs index. Richness indices (Sobs and Chao1) were also significantly negatively influenced by soil pH. Shannon and inverse Simpson indices showed that soil TP and AhN were significantly negatively correlated with diversity. The richness and diversity of AM fungi significantly decreased with increasing soil AP content and latitude. In rhizosphere soil, AM fungal richness showed a significant negative correlation with SOC and altitude but a significant positive correlation with soil AK. Longitude did not significantly influence richness or diversity in both samples (*P* > 0.05).

**TABLE 1:** Pearson correlation coefficients of soil properties and geographical variables with AM fungal α-diversity indices in the roots and rhizosphere soil of *Phoebe bournei* seedlings^*a*^

Variables	Roots				Rhizosphere soil			
	Sobs	Chao1	Shannon	inverse Simpson	Sobs	Chao1	Shannon	inverse Simpson
pH	−0.50*	−0.47*	−0.39	−0.25	−0.14	−0.2	−0.09	−0.18
TN	−0.48*	−0.42	−0.23	0.02	−0.03	−0.03	−0.21	−0.22
TP	−0.43	−0.42	−0.62**	−0.60**	0.02	−0.04	0.01	−0.17
TK	−0.08	−0.13	−0.13	−0.17	0.32	0.26	0.32	0.05
AhN	−0.39	−0.38	−0.56**	−0.54*	−0.39	−0.42	−0.22	−0.13
AP	−0.70***	−0.66**	−0.64**	−0.47*	−0.24	−0.30	−0.04	−0.07
AK	−0.19	−0.24	−0.24	−0.22	0.53*	0.50*	0.14	−0.15
SOC	−0.45*	−0.42	−0.27	−0.13	−0.56**	−0.60**	0.12	0.38
Longitude	−0.24	−0.25	0.04	0.31	0.19	0.19	−0.20	−0.28
Latitude	−0.53*	−0.53*	−0.65**	−0.58**	0.19	0.14	−0.01	−0.23
Altitude	0.34	0.33	0.18	−0.02	−0.48*	−0.45*	0.07	0.36

^*a*^ TN, total nitrogen; TP, total phosphorus; TK, total potassium; AhN, alkaline-hydrolyzable nitrogen; AP, available phosphorus; AK, available potassium; SOC, soil organic carbon. Significant correlations are shown by *P < 0.05, **P < 0.01**, and ***P < 0.001.

**Fig 6:**
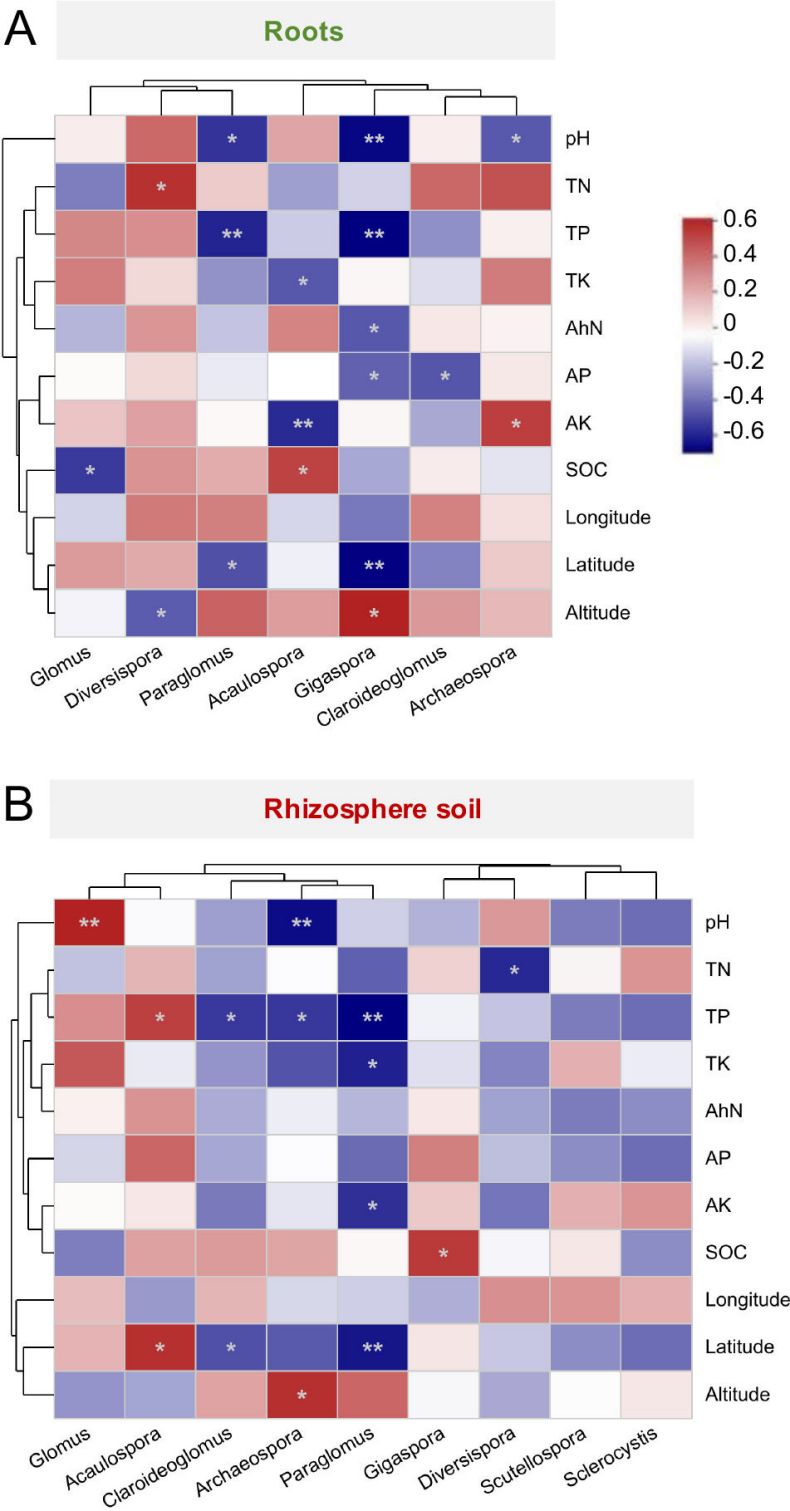
Correlations of soil properties and geographical variables with AM fungal community composition (genus level). (**A**) Roots, (**B**) rhizosphere soil of *Phoebe bournei* seedlings. * Indicates significant correlations, with significance levels of *P < 0.05 and **P < 0.01.

Spearman correlation analyses revealed that the relative abundances of AM fungal genera in roots and rhizosphere soils responded differently to soil properties and geographical variables. In roots, the abundances showed no significant correlation with longitude (*P* > 0.05; Fig. 6A). In the rhizosphere soil, the abundances showed no significant correlation with soil AhN, AP contents, and longitude (*P* > 0.05; Fig. 6B). Moreover, the relative abundance of *Scutellospora* and *Sclerocystis* in the rhizosphere soil did not statistically correlate with the soil properties and geographical variables (*P* > 0.05; Fig. 6B).

### Roles of soil properties and geographical variables in AM fungal community structure

**Fig 7:**
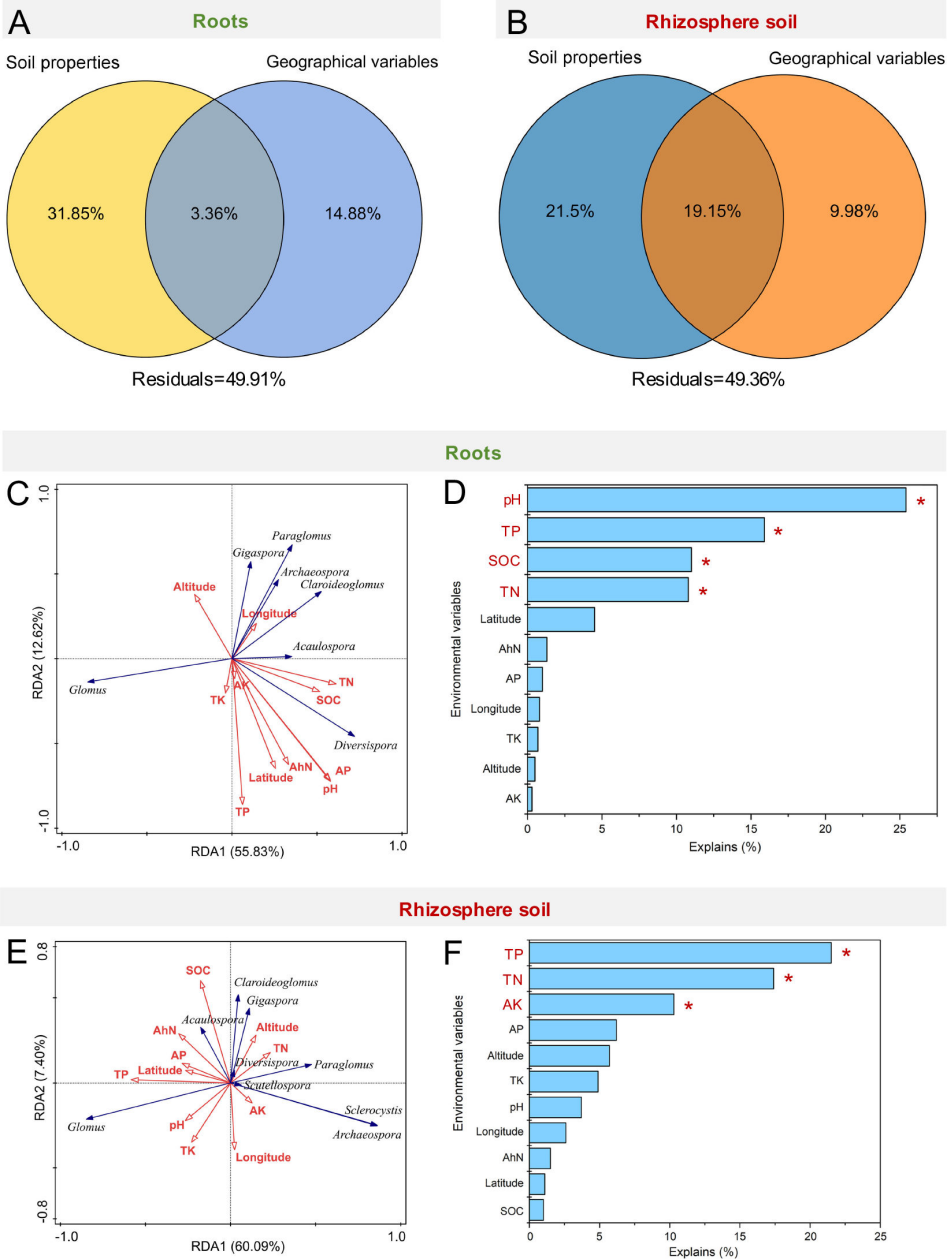
Relationships between soil properties, geographical variables, and the AM fungal community structure. VPA of soil properties, geographical variables, and the AM fungal community structure in the roots (**A**) and rhizosphere soil (**B**) of *Phoebe bournei* seedlings. (**C and E**) Redundancy analysis (RDA) of the relationships between soil properties, geographical variables (red arrows), and AM fungi genera (blue arrows) in the roots and rhizosphere soil of *Phoebe bournei*. (**D and F**) Extent to which soil properties and geographical variables explain the variations in the AM fungal community structure in the roots and rhizosphere soil. Significant correlations are shown by *P < 0.05.

A variance partitioning analysis (VPA) was performed to quantify the relative relationships of soil properties and geographical variables with AM fungal community structure. For the AM fungal community structure in the roots of *P. bournei* seedlings, while soil properties and geographical variables separately explained 31.85% and 14.88% of the total variation, their simultaneous effect accounted for 3.36% of the total variation (Fig. 7A). In the rhizosphere soil, while soil properties and geographical variables separately explained 21.50% and 9.98% of the total variation, their simultaneous effect accounted for 19.15% of the total variation (Fig. 7B). These results suggest that soil properties play major roles in shaping AM fungal community structures in the roots and rhizosphere soil of *P. bournei* seedlings.

Next, we used redundancy analysis (RDA) to identify the effects of soil properties and geographical variables on the AM fungal community structure. The first two ordination axes of RDA explained a total variation of 68.45% in the AM fungal community structure of the roots (Fig. 7C). The Monte Carlo permutation test revealed the main drivers of the AM fungal community structure in roots: pH (F = 6.5, *P <* 0.05), TP (F = 4.9, *P <* 0.05), SOC (F = 4.8, *P <* 0.05), and TN (F = 3.8, *P <* 0.05) (Fig. 7D). Regarding the rhizosphere soil, the first two ordination axes of RDA explained 67.49% of the total variation in the AM fungal community structure (Fig. 7E). The Monte Carlo permutation test revealed the main drivers of AM fungal community structure in the rhizosphere soil: TP (F = 5.2, *P <* 0.05), TN (F = 5.1, *P <* 0.05), and AK (F = 3.6, *P <* 0.05) (Fig. 7F). These findings suggest that AM fungal communities in the roots and rhizosphere soil show different responses to soil properties and geographical variables. Simultaneously, soil TP and TN—showing significant associations with the AM fungal communities in the roots and rhizosphere soil—represent the key drivers of the AM fungal community structure of *P. bournei* seedlings.

## DISCUSSION

### AM fungal community composition and co-occurrence patterns in *P. bournei* seedling roots and rhizosphere soil

This study presents the first comprehensive analysis of AM fungal communities in naturally regenerated *P. bournei* seedling roots and rhizosphere soil samples in Guizhou Province using MiSeq sequencing. A total of 918,392 high-quality sequences were obtained, which formed 305 OTUs based on the Maarj*Am* database. *Glomus* was the dominant AM fungal genus in both samples (Fig. 2C), consistent with findings from the *P. bournei* young plantation in Jiangxi Province (33). Previous studies have shown *Glomus* dominance in forest (34, 35), farmland (36), grassland (13), saline (37), and desert ecosystems (38), highlighting its wide adaptation to diverse niches.

*Glomus* dominated the rhizosphere soil and roots, while *Acaulospora* and *Claroideoglomus* were subdominant genera in the rhizosphere soil and *Diversispora* and *Paraglomus* in the roots (Fig. 2C). This difference suggests niche preferences among AM fungal taxonomic groups during their life cycles (39)—likely an important factor triggering differences in the AM fungal community composition of rhizosphere soil and roots. *Gigaspora* was present in root samples from all six locations except Shiqian, where *Diversispora* was more abundant. This could be attributed to the higher soil nutrient levels (TN, TP, AP, and AhN) in Shiqian than in the others (Fig. 1B, C, F, and G). Notably, differences in soil nutrient content may favor the growth of certain AM fungal species while contributing to the loss of others (40). Moreover, the relatively higher abundance of *Archaeospora* (Fig. 2C) observed in the rhizosphere soil samples of Rongjiang suggests that *Archaeospora* species are better adapted to lower pH and TP levels, thereby enhancing phosphorus uptake by *P. bournei* seedling roots. This finding highlights the potential value of *Archaeospora* for developing mycorrhizal inoculants, particularly for use in more acidic and lower-phosphorus soils.

In the co-occurrence network of biological communities, topological parameters such as edge number, node number, and average degree reflect network complexity (41). In this study, the co-occurrence network in the rhizosphere soil had a more complex structure (higher node and edge numbers and average degree) (Fig. 5B) than that in the roots (Fig. 5A). This may be related to the more complex community composition of AM fungi in the rhizosphere soil of *P. bournei* seedlings. A greater abundance of taxonomic members in a community indicates more interactions among them (42), thereby enhancing network complexity. Moreover, the AM fungal co-occurrence network in the rhizosphere soil showed a lower average clustering coefficient and higher negative correlations (Fig. 5B), suggesting a more robust network structure with more frequent inter-competition among the co-occurring AM fungi (43, 44).

### Effects of soil properties and geographical variables on AM fungal diversity and community composition in the roots and rhizosphere soil of *P. bournei* seedlings

AM fungi thrive in plant roots and surrounding soil, with their survival closely linked to soil conditions (4, 45). Therefore, soil properties inevitably affect their community composition and abundance. Beyond serving as a key indicator of soil nutrient availability, pH is an important factor in the nutritional function of AM fungi and their host plants (46). We found that the richness of AM fungi in *P. bournei* seedling roots was significantly negatively correlated with soil pH (Table 1), suggesting that a lower pH favors certain AM fungi taxa to colonize in *P. bournei* root systems, potentially promoting a more efficient absorption of nutrients.

We found that declines in AM fungal diversity and richness were related to high levels of soil P and N (Table 1), which the “trade equilibrium model” explains (47). According to this model, as soil P and N levels increase, the host plant’s carbon investment in its AM fungal partners decreases. This reduction may intensify carbon competition among AM fungi, ultimately lowering their diversity and richness. Meanwhile, the significant negative associations between SOC content and AM fungal richness in rhizosphere soil and roots suggest that higher SOC content is linked to lower abundance and potentially reduced functional activity of AM fungal species. A similar inverse relationship between AM fungal richness and SOC was previously observed in the soils of the Daqingshan Mountains in China (48) and in the roots of *Triticum aestivum* L. (49). Additionally, accumulated soil AK appeared to stimulate the increases of AM fungal species in the *P. bournei* rhizosphere soil microenvironment.

We also found a strong correlation between soil nutrient levels and the relative abundance of certain AM fungal genera in the rhizosphere soil and roots of *P. bournei* seedlings (Fig. 6). Soil nutrient contents were negatively correlated with *Paraglomus* and *Claroideoglomus* relative abundances, while they were either positively or negatively correlated with *Glomus*, *Diversispora*, *Acaulospora*, *Gigaspora*, and *Archaeospora* relative abundances. These findings agree with earlier studies indicating that different AM fungal taxa exhibit differences in sensitivity and strategies to variations in soil nutrient levels (50, 51). These differences may result in shifts in the AM fungal diversity and community composition, as observed in the present study.

Beyond soil properties, geographical variables also influenced the AM fungal community. The current study found a significant negative correlation between latitude and the diversity and richness indices of AM fungi in *P. bournei* seedling roots (Table 1). This deviated from Jiang et al.’s study (15), which showed higher AM fungal diversity and richness at higher latitudes in the rhizosphere soil of litchi and mango orchards. This inconsistency may have resulted from investigations of different ecosystems, host plants, and niches. Moreover, the relative abundance of *Paraglomus*, *Gigaspora*, and *Claroideoglomus* showed a significantly negative correlation with latitude, whereas the opposite trend was observed for *Acaulospora* (Fig. 6). These results suggest that AM fungi exhibit distinct distribution patterns with latitude. Furthermore, altitude had a significantly negative effect on AM fungal richness in the rhizosphere soil of *P. bournei* seedlings (Table 1). This finding, consistent with a previous study, suggests that AM fungal richness decreases with increasing altitude (17). Nevertheless, *Gigaspora* in the roots and *Archaeospora* in the rhizosphere soil showed significant positive correlations with altitude, indicating their greater tolerance and adaptability at higher altitudes with lower temperatures and precipitation.

### Drivers of AM fungal community compositions and structures

VPA and RDA revealed soil properties as the major drivers of variations in AM fungal community structures in the rhizosphere soil and roots of *P. bournei* seedlings. Moreover, soil TN and TP were simultaneously and significantly connected with variations in AM fungal community structures in both samples (Fig. 7D and F) and thus were identified as the key drivers shifting such structures. Previous studies have emphasized the role of soil phosphorus in influencing AM fungal community structure (22, 35). In the rhizosphere soil, soil phosphorus can affect such structure by stimulating hyphal growth, spore distribution, and germination (52). Roots uptake phosphorus directly from the soil via root epidermal cells and root hairs when the soil phosphorus supply is sufficient (53), which may reduce reliance on AM fungi. However, roots enhance symbiosis with AM fungi to facilitate phosphorus uptake under conditions of scarcity (54), thereby promoting hyphal growth while increasing AM fungal biomass, resulting in altered compositions and structures of AM fungal communities.

The sensitivity of AM fungal taxa to N varies (55), potentially leading to diverse responses to different N concentrations and alterations in the AM fungal community structure. For example, N deficiency could directly inhibit AM fungal growth (56). However, N addition might cause alterations in the AM fungal community structure by inducing shifts in the relative abundance of some AM fungal taxa or increasing competition for host niches and/or for nutrients among AM fungal taxa (57, 58). Moreover, N enrichment accounted for changes in AM fungal community structure and induced the mycorrhizal functioning of AM fungi toward parasitism (7). This suggests that changes in soil N can indirectly regulate AM fungal community structure by altering host plant–AM fungus interactions. These features may explain why soil TP and TN were key drivers of AM fungal community structures in the studied roots and rhizosphere soil of naturally regenerated *P. bournei* seedlings.

### Conclusion

This study revealed the diversity, community composition, and co-occurrence network structure of AM fungi in the roots and rhizosphere soil of naturally regenerated *P. bournei* seedlings in Guizhou, subtropical China. Soil properties were identified as the main drivers of changes in AM fungal community structure, with total P and total N serving as key drivers for variations in such structures associated with the roots and rhizosphere soil of naturally regenerated *P. bournei* seedlings. Our study not only provides a better understanding for further exploring the applications of AM fungal species in the cultivation of *P. bournei* seedlings and afforestation, but also offers valuable insight into the structuring of *P. bournei*–AM fungi symbiotic systems in certain special environments.

## MATERIALS AND METHODS

### Study area

The investigations were conducted in Guizhou Province, a subtropical region of southwestern China (26°2′–27°26′N, 107°2′–109°10′E). Seven *P. bournei* natural communities were selected as sampling locations: Liping (LP), Rongjiang (RJ), Taijiang (TJ), Shiqian (SQ), Leishan (LS), Yuqing (YQ), and Huishui (HS) (Fig. S3; Table S2). These areas have four distinct seasons and abundant precipitation, as is typical of a subtropical humid monsoon climate. The mean annual temperature and precipitation in the seven areas were 14.5°C–16.8°C and 1,135–1,289 mm, respectively, and the altitude was 402–999 m (Table S2).

### Sample collection and preparation

Root and rhizosphere soil samples of *P. bournei* were collected from each location in July 2020. At each location, three healthy *P. bournei* adult trees with a straight-line distance of >15 m from each other were randomly selected to ensure sample independence. Under the canopy of each adult tree, 12 naturally regenerated *P. bournei* seedlings (3 plants × 4 different directions) of similar growth status were selected as the research objects. The selected seedlings were carefully excavated with the whole root system. Bulk soil was gently removed by shaking the seedlings, and the soil adhering to the root surface was reserved as the rhizosphere soil. Fine roots were detached from multiple positions of the root system. Rhizosphere soil or fine roots from the 12 seedlings were well-pooled together into a composite sample for the rhizosphere soil or roots. All composite root and rhizosphere soil samples were placed in plastic sterile bags separately, labeled, stored at 4°C, and returned to the laboratory. Finally, 21 rhizosphere soil samples and 21 root samples were obtained.

In the laboratory, the collected fine roots were rinsed with distilled water repeatedly, frozen in liquid nitrogen, and then stored in an ultra-cold storage freezer at −80°C until DNA extraction. Each rhizosphere soil sample was divided into two parts. One part was stored under the same conditions as the root samples before DNA extraction. Another part was air-dried and then screened to determine its soil properties.

### Soil properties analysis

Soil pH value was measured in a soil/water suspension (wt/vol, 1:2.5) with a pH meter (Mettler-Toledo FE 28, Shanghai, China). TN was determined using the Kjeldahl method. TK and TP were digested using H_2_SO_4_–HClO_4_ and determined by flame photometry and the molybdenum–antimony colorimetric method, respectively. SOC was determined using K_2_Cr_2_O_7_ oxidation–reduction titration. AhN was determined by the NaOH hydrolysis diffusion method. AP was determined with HCl–H_2_SO_4_ extraction colorimetry. AK was assessed by flame photometry after samples were extracted with a 1 M NH_4_OAc solution (pH = 7.0). These determinations were conducted using standard procedures (59).

### AM fungal DNA extraction, nested PCR, and Miseq sequencing

Total genomic DNA was separately extracted from frozen soil and roots using the Fast DNA Spin Kit (MP Biomedicals, CA, USA) following the manufacturer’s instructions. DNA concentration and quality were verified with a NanoDrop-2000 Spectrophotometer (Thermo Scientific, USA) and 1.0% agarose gel electrophoresis, respectively. A nested PCR strategy was employed to amplify the gene fragments of AM fungal 18S rRNA. The primers AML1/AML2 (60) were used in the first runs, whereas AMV4.5NF/AMDGR (61) were used in the second runs. PCRs were conducted in a total of 20 µL mixture system. The specific amplification system components and cycling conditions were performed following those reported previously (62), except that the amplification cycle numbers were 32 cycles for the first round and 30 cycles for the second round. Three PCR replicates were performed for each sample and then mixed to generate a composite PCR product. After being detected on a 2% agarose gel, these composite PCR products were purified and then equimolarly pooled. Later, the purified amplicons were sent to Majorbio Bio-Pharm Technology Co., Ltd. (Shanghai, China) for paired-end sequencing (2 × 300 bp) on an Illumina MiSeq PE300 platform.

### Data analysis

The raw sequence data were de-multiplexed, then quality-filtered by Fastp (v0.19.6), and merged by FLASH (v1.2.11). Subsequently, the remaining high-quality sequences were grouped into different OTUs using Usearch (v11.0) (63) with 97% sequence similarity. The most abundant sequence from each OTU was selected as the representative sequence. Representative sequences of OTUs were taxonomically identified by blasting against the Maarj*AM* database (64). The α-diversity metrics (Sobs, Chao1, Shannon, inverse Simpson index, and Good’s coverage) were estimated using Mothur (v1.30.2) after rarefying samples to equal sequence reads (the lowest sequencing depth among all samples, 17,793 reads).

Statistical analyses were undertaken using SPSS 26.0 (SPSS Inc., Armonk, NY, USA) or R version 4.0.2 (R Core Team, 2020; https://www.r-project.org/). Analysis of differences in soil properties was conducted using the nonparametric Kruskal–Wallis rank sum test. To demonstrate the distribution of unique and shared OTUs between the root and rhizosphere soil samples, a Venn diagram was generated by the VennDiagram package of R (65). The relative abundances of AM fungal communities at different taxonomic levels were calculated and visualized using the ggplot2 package of R (66). After Shapiro–Wilk normality tests confirmed that the data sets followed a normal distribution, a one-way analysis of variance and Tukey’s multiple range test (*P* < 0.05) by SPSS (v26.0) were performed to compare AM fungi α-diversity. PCoA based on Bray–Curtis distances was performed to evaluate and visualize variations in AM fungal community composition among sampling locations using the vegan package of R (67). Additionally, PERMANOVA was performed using the “adonis” function of the R package vegan (67, 68) to verify the significant dissimilarities among sampling locations.

Co-occurrence networks were applied to realize the AM fungal interrelationships individually for the rhizosphere soil and roots based on Spearman’s correlation coefficients. To avoid the bias introduced by different OTU numbers, OTUs with relative abundances (<0.1%) for each group were removed from the network analysis. Networks of significant correlations (|r| > 0.5, *P* < 0.05) and several network topological parameters were visualized and calculated using the Fruchterman–Reingold layout on the Gephi platform (v0.10.1). Pearson’s correlation analysis was conducted to express the relationships between soil properties, geographical variables, and α-diversity of AM fungi. The associations of soil properties and geographical variables with AM fungal community composition were assessed by Spearman’s correlation analysis, and a heat map was constructed using the pheatmap package in R (69). VPA for exploring how soil properties and geographical variables contributed to the variation of AM fungal community structures (at the OTU level) was performed using the “varpart” function of the vegan package in R (67, 70). RDA with Monte Carlo permutation tests (999 permutations) was executed using CANOCO 5.0 (Microcomputer Power, Ithaca, NY, USA) to assess the influence of soil properties and geographical variables on AM fungal community structures (at the genus level).

## Data Availability

The raw data obtained from MiSeq sequencing have been imported into the Sequence Read Archive database of the National Center for Biotechnology Information (BioProject PRJNA1182106).

## SUPPLEMENTAL MATERIAL

Supplemental figures and tables spectrum.00210-25-s0001.pdf Figures of rarefaction curves, Venn, and sampling locations. Tables of AM fungal sequences information, and geographical location and climate of seven sampling locations.

ASM does not own the copyrights to Supplemental Material that may be linked to, or accessed through, an article. The authors have granted ASM a non-exclusive, world-wide license to publish the Supplemental Material files. Please contact the corresponding author directly for reuse.
