# Supplementary material for: Composition and driving factors of arbuscular mycorrhizal fungal communities in the roots and rhizosphere soil of naturally regenerated Phoebe bournei seedlings in Guizhou Province, China
Source: Microbiol Spectr. 2025 Jul 7;13(8):e00210-25. doi: 10.1128/spectrum.00210-25 (PMC12323378; doi:10.1128/spectrum.00210-25)
Supplement: Supplemental figures and tables — Figures of rarefaction curves, Venn, and sampling locations. Tables of AM fungal sequences information, and geographical location and climate of seven sampling locations. [file spectrum.00210-25-s0001.pdf]

Supplementary Material

Composition and driving factors of arbuscular mycorrhizal fungal communities in the roots and rhizosphere soil of naturally regenerated *Phoebe bournei* seedlings in Guizhou Province, China

Xian Liang[a,b], Xinyuan Lu[a,b], Yi Wei[a,b], Fuyin Jiang[a,b], Mingbin Wang[a,b], Xiaoli Wei[a,b] #

[a]College of Forestry, Guizhou University, Guiyang 550025, China

[b]Institute for Forest Resources and Environment of Guizhou, Guizhou University, Guiyang 550025, China

# Address correspondence to Xiaoli Wei, gdwxl2022@163.com

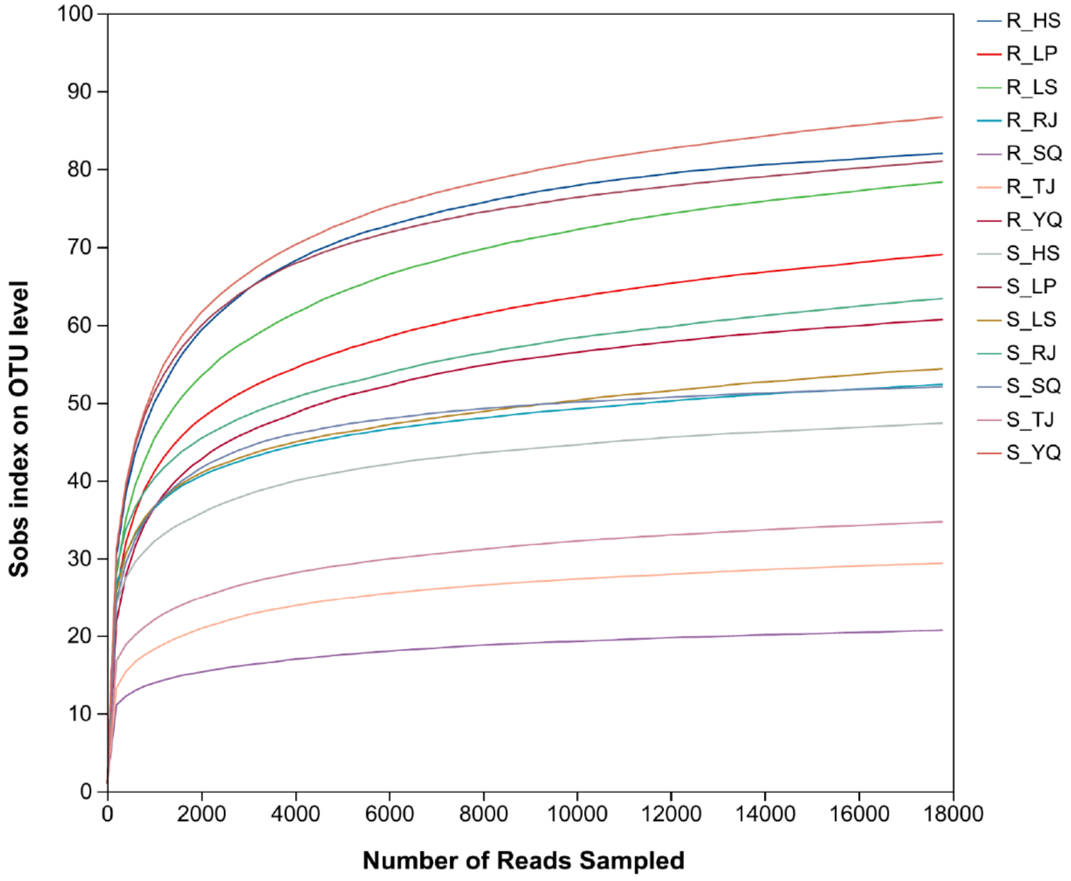

Figure S1. Rarefaction curves for the observed OTUs of AM fungi detected in *Phoebe bournei* seedlings roots and rhizosphere soil from different sampling locations. R: root samples; S: rhizosphere soil samples.

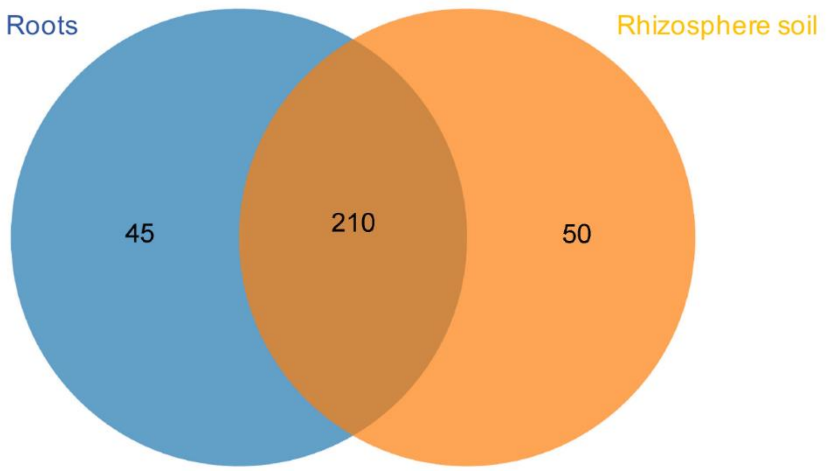

Figure S2. Venn diagram of AM fungal OTUs in the *Phoebe bournei* seedlings. Numbers represent unique and shared OTUs from roots and rhizosphere soil, respectively.

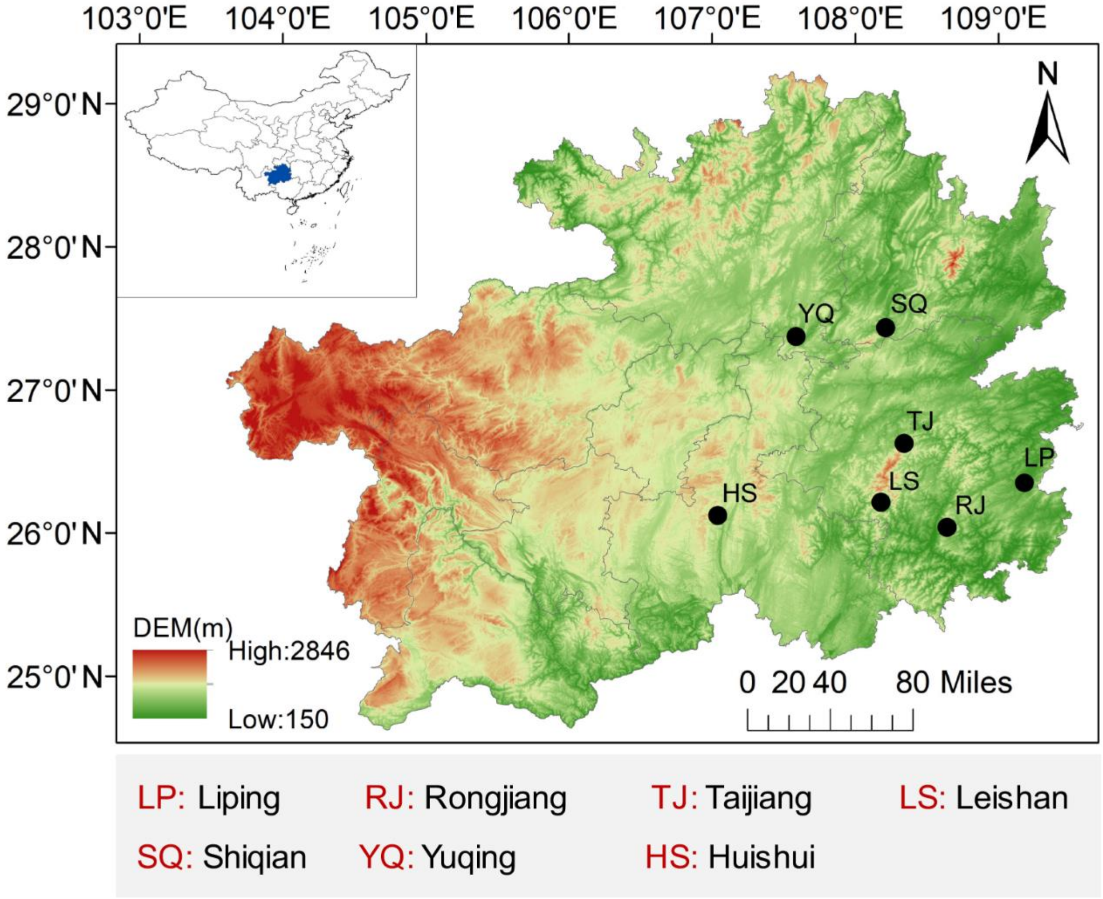

Figure S3. Root and rhizosphere soil sampling locations of *Phoebe bournei* seedlings.

Table S1 AM fungal sequences, mean sequence length, and coverage in root and rhizosphere soil samples

Samples	Roots			Rhizosphere Soil		
	Sequence	Mean length	Good's Coverage	Sequence	Mean length	Good's Coverage
LP-1	21735	215.76	0.9994	21599	215.93	0.9993
LP-2	22403	215.90	0.9996	22164	216.23	0.9997
LP-3	21799	215.84	0.9993	21442	216.24	0.9996
RJ-1	21990	216.23	0.9996	20013	215.85	0.9993
RJ-2	22877	216.06	0.9999	21819	216.12	0.9998
RJ-3	21560	216.08	0.9995	18832	216.80	0.9994
TJ-1	22192	216.42	0.9997	23884	216.59	0.9998
TJ-2	21554	216.33	0.9999	22172	217.56	0.9998
TJ-3	22976	216.07	0.9998	22035	216.59	0.9998
SQ-1	22099	217.19	0.9999	23085	216.10	0.9998
SQ-2	21592	216.55	0.9998	22364	216.11	0.9997
SQ-3	22221	215.54	0.9999	23014	216.16	0.9999
LS-1	22667	216.00	0.9994	21659	215.42	0.9996
LS-2	22088	216.04	0.9994	17793	216.73	0.9994
LS-3	22812	215.97	0.9995	21648	216.01	0.9997
YQ-1	22956	215.99	0.9998	22915	215.77	0.9995
YQ-2	21994	215.96	0.9996	22067	216.15	0.9993
YQ-3	22630	215.89	0.9994	20486	215.96	0.9996
HS-1	21314	215.82	0.9994	21845	215.86	0.9996
HS-2	22499	215.94	0.9997	21533	216.33	0.9999
HS-3	20602	216.04	0.9998	21463	215.99	0.9997

Table S2 Geographical location and climate of seven sampling locations

Sampling sites	Longitude (E)	Latitude (N)	Altitude (m)	Precipitation (mm/year)	Annual mean temperature (°C)
LP	109.179081°	26.349497°	402	1289	16.8
RJ	108.639894°	26.035736°	766	1284	15.7
TJ	108.340006°	26.626178°	852	1229	14.5
SQ	108.211503°	27.433328°	515	1158	15.9
LS	108.178461°	26.211933°	951	1250	15.4
YQ	107.586272°	27.371353°	685	1135	16.4
HS	107.039839°	26.118683°	999	1192	15.7
